# Accurate prediction of DNA N^4^-methylcytosine sites via boost-learning various types of sequence features

**DOI:** 10.1186/s12864-020-07033-8

**Published:** 2020-09-11

**Authors:** Zhixun Zhao, Xiaocai Zhang, Fang Chen, Liang Fang, Jinyan Li

**Affiliations:** 1 Advanced Analytics Institute, Faculty of Engineering and Information Technology, University of Technology Sydney, PO Box 123, Broadway, Sydney, NSW 2007 Australia; 2 Data Science Institute, University of Technology Sydney, PO Box 123, Broadway, Sydney, NSW 2007 Australia; 3 School of Computer, National University of Defense Technology, Changsha, 410073 China

**Keywords:** DNA N4-methylcytosine, Sequence feature, Feature selection, Site prediction

## Abstract

**Background:**

DNA N4-methylcytosine (4mC) is a critical epigenetic modification and has various roles in the restriction-modification system. Due to the high cost of experimental laboratory detection, computational methods using sequence characteristics and machine learning algorithms have been explored to identify 4mC sites from DNA sequences. However, state-of-the-art methods have limited performance because of the lack of effective sequence features and the ad hoc choice of learning algorithms to cope with this problem. This paper is aimed to propose new sequence feature space and a machine learning algorithm with feature selection scheme to address the problem.

**Results:**

The feature importance score distributions in datasets of six species are firstly reported and analyzed. Then the impact of the feature selection on model performance is evaluated by independent testing on benchmark datasets, where ACC and MCC measurements on the performance after feature selection increase by 2.3% to 9.7% and 0.05 to 0.19, respectively. The proposed method is compared with three state-of-the-art predictors using independent test and 10-fold cross-validations, and our method outperforms in all datasets, especially improving the ACC by 3.02% to 7.89% and MCC by 0.06 to 0.15 in the independent test. Two detailed case studies by the proposed method have confirmed the excellent overall performance and correctly identified 24 of 26 4mC sites from the C.elegans gene, and 126 out of 137 4mC sites from the D.melanogaster gene.

**Conclusions:**

The results show that the proposed feature space and learning algorithm with feature selection can improve the performance of DNA 4mC prediction on the benchmark datasets. The two case studies prove the effectiveness of our method in practical situations.

## Background

As an essential epigenetic modification, DNA base methylation expands the DNA content and plays crucial roles in regulating various cellular processes [1–3]. According to the location where a methylated group occurs in the DNA sequence, there are many kinds of DNA base methylation. For example, 5-Methylcytosine (5mC), N6-methyladenine (6mA) and N4-methylcytosine (4mC) are the most common types [4–6]. 5mC occurs at the C5-position of cytosine and is the dominant methylation type in eukaryotic genomes, actively involved in differentiation, gene expression, genomic imprinting, preservation of chromosome stability, aging, suppression of repetitive element, and X chromosome inactivation [7–10]. In prokaryotes, 6mA and 4mC constitute the majority of DNA base methylations [11]. 6mA occurs at the N6-position of adenine and is a marker in gene regulation, development, DNA replication, repair, and expression [12–14]. 4mC happens at the N4-amino group of cytosine and participates in the restriction-modification system that provides a bacterial immune response against occupying DNA, DNA repair, expression, or replication [15–17]. Compared with the studies for 5mC and 6mA, biological functions of 4mC are much investigated due to the lack of sufficient detection methods.

The precise location of the DNA base methylation was a hard problem in the past for a long time. It is not affordable to locate the DNA 5mC on a large scale until the whole-genome bisulfite sequencing, and the next-generation sequencing techniques were developed [18, 19]. The detection of 6mA and 4mC in the level of whole-genome became available after the single-molecule real-time sequencing (SMRT) technology was introduced [4, 20]. Then a next-generation sequencing method called 4mC-Tet-assisted-bisulphite-sequencing and another method based on engineered transcription-activator like effectors were developed for 4mC identification [21]. However, the experimental methods were of high cost and cannot identify 4mC sites with time efficiency. Recently, the rapid development of machine learning algorithms provides a promising computational approach to address classification problems in bioinformatics, and researchers have explored using computational methods to identify 4mC sites from DNA sequences.

Collecting data from public SMRT sequencing experiments, Ye *et al* built the first DNA 6mA and 4mC database named MethSMRT for 156 species [22]. Chen *et al* [23] proposed an SVM based prediction model iDNA4mC using the nucleotide chemical property and sequential nucleotide frequency features. Recently, 4mCPred and 4mcPred-SVM were developed to improve the site prediction performance [24,25]. In 4mCPred, He *et al* used two new features PSTNP and EIIP with a simple feature selection. Wei *et al* built 4mcPred-SVM with four kinds of sequence features and a two-step feature optimization. Recently, some other predictors have been developed to identified 4mC site in the DNA sequence for Mouse [26,27], Escherichia coli [28], Rosaceae [29] and so on [30,31].

The core idea of the previous research is to transform 4mC-contained DNA sequences into various kinds of features as the input of the machine learning algorithms.However, these features are not adequate to make the prediction methods to achieve excellent performance. Through the analysis on the sequence logos, we observe that the adjacent nucleotides’ characteristics are potentially essential. Thus we extract the contiguous nucleotides sequence characteristics like k-nucleotide frequency, k-spectrum nucleotide pair frequency, and PseDNC as features to describe the sequences. Besides, two global sequence features, one-hot binary and sequential nucleotide frequency, are also merged into our feature space. As global features have the complete information of DNA sequence and the local features can underline specific sequence patterns, the combined feature space is highly expected to improve the prediction performance.

Since feature selection can reduce the feature space dimension and the modelling complexities [32], the existing 4mC prediction methods, including 4mCPred and 4mCPred_SVM, employed a feature selection scheme based on the F-score and sequential forward search (SFS) strategy [24,25]. Although the F-score can evaluate the feature importance according to the relevance between the feature and label, the performance of the selected feature subset was still under-realized. In this paper, we propose an embedded feature selection scheme, in which features are ranked with the feature importance scores derived by the XGBoost classifier training process. Supported by information entropy theory, the feature importance here is more meaningful than F-score. Then lower-ranked features are removed one by one, each round with a cross-validation assessment on the performance of the selected feature subset.

**Fig. 1:**
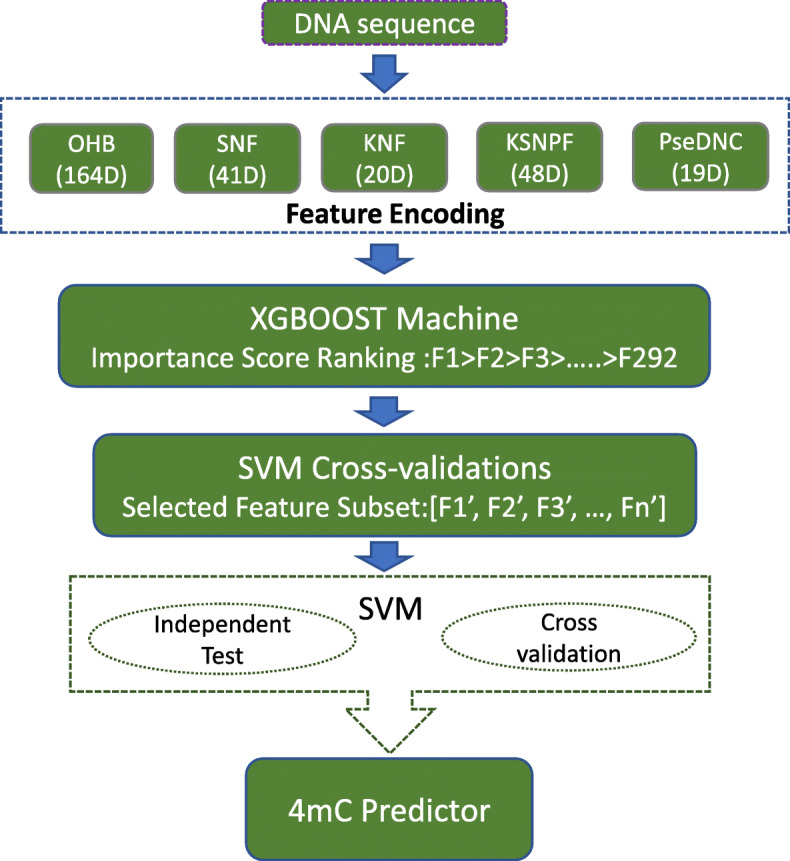
Framework of proposed model construction

The flowchart of our approach is shown in Fig. 1 where the new sequence feature space and feature selection scheme are depicted for DNA 4mC site prediction. First, the DNA sequence is encoded into five kinds of features, a total of 292 dimensions. Second, an XGBoost machine is trained and the feature importance scores from the training are used to rank all the features. Last, an SVM-based prediction model is built, and the parameters are optimized with 10-fold cross-validation.

In the results section, we analyze the feature importance in our feature space and show that feature selection improves the model performance significantly in the independent test. Besides, we compare the proposed method with three state-of-art methods, iDNA4mC, 4mCPred, and 4mCPred_SVM in independent test and 10-fold cross-validation on benchmark datasets, and the proposed method achieves much better performance. Two detailed case studies for 4mC site prediction on the dlk-1 and DSCAM genes partly prove the effectiveness of our approach in practical situations.

## Results

This section reports the feature importance scores obtained from the XGBoost machine and analyzes the influence of the feature selection on prediction performance. Then three state-of-the-art predictors are compared with the proposed method in the independent test and 10-fold cross-validation on benchmark datasets. At last, we present results from two case studies which were conducted to identify the 4mC sites in the C.elegans and D.melanogaster genes.

### Feature importance analysis

**Fig. 2:**
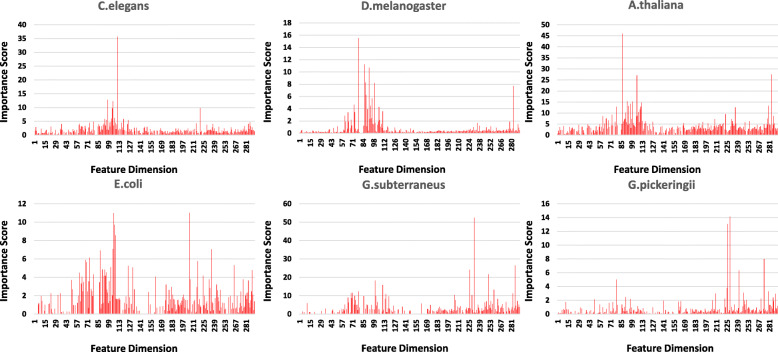
Sequence feature importance distribution

As stated, five types of sequence features are created to constitute a 292-dimensional feature space. Among the 292 dimensions, OHB is from D1 to D164; SNF is from D165 to D205; KNF is from D206 to 225; KSNPF is from D226 to 273 and PseDNC is from D274 to D292. The feature importance scores are obtained from the training process of the XGBoost machine. The importance score distributions for all the datasets are illustrated in Fig. 2. Top 30 feature dimensions are reported in Table S2 of Additional File 1 and feature importance scores of all the feature dimensions are in Additional File 2.

It is understood that each feature dimension has distinct importance scores in different species. OHB and PseDNC features have relatively high average scores in all species. In particular, OHB features have the highest average score in C.elegans, D.melanogaster and A.thaliana. KSNPF feature not only gets a high importance score in A.thaliana, E.coli and G.subterraneus like KNF features, but also has the highest average score in G.pickeringii. SNF feature just stands out in E.coli. The features’ importance score ranges from 0 to 50 and some feature dimensions’ scores are such low that they are less important in the classification and may have noise effects on model performance. Thus, the feature selection before the training is potentially useful to improve model accuracy.

### Impact of feature selection on classification

**Fig. 3:**
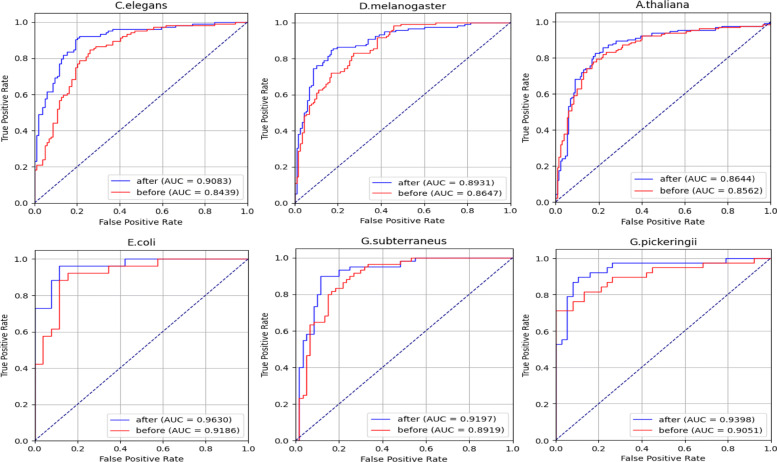
The ROC curves before and after feature selection

We first evaluate the model performance via independent test without feature selection before model training. Then the independent test is carried out with feature selection, where the benchmark datasets divisions and SVM parameters are kept the same. Table 1 and Fig. 3 show the independent test performance before and after feature selection.

**Table 1:** The independent test performance before and after feature selection(Sn, Sp and ACC:%)

Datasets	Selection	Sn	Sp	ACC	MCC
C.elegans	before	82.69	75.00	78.85	0.58
	after	94.23	78.85	86.53	0.74
D.melanogaster	before	74.57	77.12	75.85	0.52
	after	84.74	86.44	85.59	0.71
A.thaliana	before	82.57	76.51	79.54	0.59
	after	80.30	83.33	81.81	0.64
E.coli	before	92.30	69.23	80.76	0.63
	after	88.46	88.46	88.46	0.77
G.subterraneus	before	83.33	75.00	79.17	0.59
	after	91.67	81.67	86.67	0.74
G.pickeringii	before	81.57	78.94	80.26	0.61
	after	86.84	89.47	88.15	0.76

**Fig. 4:**
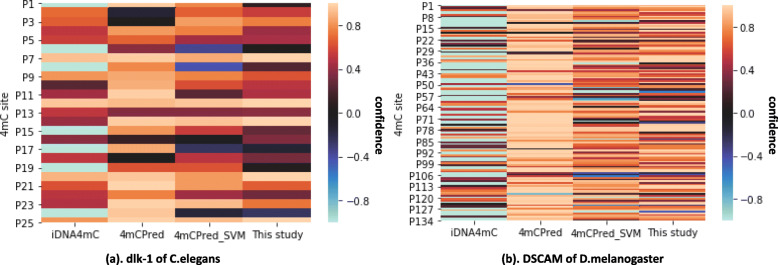
The confidence of predicted label in case studies

The independent test after feature selection improves the model performance in all the species. In C.elegans, feature selection improved Sn, Sp, ACC and MCC by 7.54%, 3.85%, 7.74% and 0.16. In D.melanogaster, the model performance has the most considerable improvement by 10.17%, 9.32%, 9.74% and 0.19 for Sn, Sp, ACC and MCC, respectively. For A.thaliana, Sp increased by 6.82% while ACC and MCC slightly increased by 2.27% and 0.05. Besides, Sp, ACC and MCC improved by 9.23%, 7.7% and 0.14 in E.coli dataset. In G.subterraneus, the metrics improvement is by 8.34% for Sn, 6.67% for Sp, 7.5% for ACC and 0.15 for MCC. As for G.pickeringii, the performance is improved by 5.17%, 10.73%, 7.89% and 0.15 in terms of Sn, Sp, ACC and MCC with feature selection. From Fig. 4, it’s obvious that the AUCs after feature selection become better in all the species. The most massive AUC growth exists in C.elegans by 0.06 and the least growth is by 0.01 in A.thaliana. The results imply that the proposed feature selection scheme enhances the performance of the SVM model by selecting effective features from the original feature space.

### Comparison with state-of-the-art predictors

Three state-of-the-art DNA 4mC prediction methods, iDNA4mC, 4mCPred, and 4mCPred_SVM are compared with the proposed method. The comparison was conducted using the independent test and cross-validation test on the benchmark datasets.

The independent test results by iDNA4mC and 4mCPred were reported in [24], and we cannot find the independent test results of 4mCPred_SVM method. Since 4mCPred_SVM only provides the final prediction model, it’s not available to rebuild the independent test. Thus, here we compare our method with iDNA4mC and 4mCPred in independent test under the same division of training and testing data. The results of independent test are presented in Table 2. Oour method outperforms the other methods in all species. Generally, the proposed method improves ACC from 3.02% to 7.89% and increases MCC from 0.06 to 0.15. Especially, a significant improvement of our approach can be observed in G.pickeringii (improving Sn by 5.26%, Spby 10.52%, ACC by 7.89%, and MCC by 0.15).

**Table 2:** Independent Test Results on Benchmark Datasets (Sn, Sp and ACC:%)

Methods	Datasets	Sn	Sp	ACC	MCC
	C.elegans	80.77	73.08	76.92	0.54
	D.melanogaster	74.58	77.97	76.27	0.53
iDNA4mC	A.thaliana	80.3	77.27	78.79	0.58
	E.coli	96.15	69.23	82.69	0.68
	G.subterraneus	85.00	76.67	80.83	0.62
	G.pickeringii	81.58	78.95	80.26	0.61
	C.elegans	85.58	78.85	82.21	0.65
	D.melanogaster	83.90	81.36	82.63	0.65
4mCPred	A.thaliana	76.52	76.52	76.52	0.53
	E.coli	84.62	80.77	82.69	0.65
	G.subterraneus	91.67	75.00	83.33	0.68
	G.pickeringii	86.84	68.42	77.63	0.56
	C.elegans	94.23	78.85	86.53	0.74
	D.melanogaster	84.74	86.44	85.59	0.71
this	A.thaliana	80.30	83.33	81.81	0.64
study	E.coli	88.46	88.46	88.46	0.77
	G.subterraneus	91.67	81.67	86.67	0.74
	G.pickeringii	86.84	89.47	88.15	0.76

We performed a 10-fold cross-validation with the same process as the existing methods. The cross-validation results of the three state-of-the-art predictors were reported in the publication of 4mCPred_SVM [25], where the reported performance of 4mCPred has been modified by solving the over-estimated problem. The summary of cross-validations are illustrated in Table 3. Except for the four evaluation metrics, we also list the sample count of TP (True Positive), FN (False Negative), FP (False Positive) and TN (True Negative). As shown in the table, in D.melanogaster, A.thaliana and Gpickeringii, our method has the most TP and TN counts, increasing ACC by 0.7% to 1.7% and MCC by 0.015 to 0.033. In G.subterraneus, our method has the highest TN, improving more ACC and MCC by 1% and 0.02% than 4mC_SVM which has the second-best performance. Additionally, the TP and TN of our method are not the highest in C.elegans and E.coli, but our method slightly improve the ACC and MCC by 1% and 0.02 in E.coli and has a comparative performance with 4mCPred, better than other two methods in C.elegans.

**Table 3:** Cross Validation Result on Benchmark Datasets (Sn, Sp and ACC:%; TP: true positive, FN: false negative, FP: false positive, TN: true negative)

Datasets	Methods	Sn	Sp	ACC	MCC	TP	FN	FP	TN
	iDNA4mC	79.7	77.5	78.6	0.572	1328	316	349	1205
C.elegans	4mCPred	82.5	82.6	82.6	0.652	1282	272	270	1284
	4mCPred_SVM	82.4	80.7	81.5	0.631	1280	274	300	1254
	this study	84.9	80.4	82.6	0.653	1319	235	305	1249
	iDNA4mC	83.3	79.1	81.2	0.625	1474	295	369	1400
D.melanogaster	4mCPred	82.4	82.1	82.2	0.646	1458	311	317	1452
	4mCPred_SVM	83.8	82.2	83.0	0.661	1483	286	314	1455
	this study	85.4	83.2	84.3	0.686	1510	259	297	1472
	iDNA4mC	75.7	76.2	76.0	0.519	1498	480	471	1507
A.thaliana	4mCPred	75.5	78.0	76.8	0.536	1494	484	435	1543
	4mCPred_SVM	77.8	79.6	78.7	0.573	1538	440	404	1574
	this study	78.3	80.5	79.4	0.589	1549	429	385	1593
	iDNA4mC	82.0	77.8	79.9	0.598	318	70	86	302
E.coli	4mCPred	81.9	83.2	82.6	0.655	318	70	65	302
	4mCPred_SVM	85.8	80.7	83.3	0.666	333	51	67	321
	this study	86.1	82.5	84.3	0.686	334	54	68	320
	iDNA4mC	82.2	80.8	81.5	0.630	745	161	174	732
G.subterraneus	4mCPred	81.8	83.7	82.8	0.662	742	164	148	758
	4mCPred_SVM	84.0	83.4	83.7	0.674	760	145	150	755
	this study	83.6	85.7	84.7	0.694	757	148	129	776
	iDNA4mC	82.4	83.8	83.1	0.663	469	100	92	477
G.pickeringii	4mCPred	85.0	81.0	83.0	0.668	484	85	108	461
	4mCPred_SVM	86.3	85.8	86.0	0.721	491	78	81	488
	this study	86.3	89.1	87.7	0.754	491	78	62	507

It’s clear that our method achieves better overall performance than the existing predictors in independent and cross-validation tests. The improvement of ACC indicates that our method accurately identifies more 4mC sites and the increase of MCC means that our method has more balanced performance for classifying positive and negative samples. Therefore, our method is more effective to identify DNA 4mC sites than the existing predictors.

### Case studies

To confirm the effectiveness of our method to solve practical problems, two detailed case studies are conducted. C.elegans and D.melanogaster are model organisms widely applied in human disease-related research works, like Parkinson and human aging research investigations [33–36]. As 4mC plays critical roles in DNA expression and replication in these models, we describe how our method can help identify 4mC sites more accurately in the related genes. We focus on the dlk-1 gene which can promote mRNA stability and local translation in C.elegans [37], and on DSCAM gene which can contribute to the specificity of neuronal connectivity in D.melanogaster [38].

The 26 and 137 validated 4mC sites in dlk-1 and DSCAM gene are collected from the MethSMRT database. The collected 4mC-contained DNA sequences are all 41-bit, that can be directly submitted into the web tools of three state-of-the-art methods. The prediction result are depicted in Fig. 4 and Table 4. Figure 4 shows the label confidence predicted by these four predictors, where the positive confidence refers that the corresponding site is predicted to be 4mC site and the negative confidence means the site is predicted to be a non-4mC site. As shown in the figure, iDNA4mC achieves the worst performance in both two cases, and half of the predictions are incorrect in DSCAM gene. 4mCPred, 4mCPred_SVM and the proposed method have similar performance in the DSCAM gene case, while the results made by 4mCPred and our proposed method on the dlk-1 gene are better than 4mCPred_SVM.

**Table 4:** 4mC site identificaiton in case studies (TP: True Postive,; FN: False Negative)

Case	Methods	Total	TP	FN
	iDNA4mC	26	19	7
dlk-1	4mCPred	26	25	1
	4mCPred_SVM	26	20	6
	This study	26	24	2
	iDNA4mC	137	70	67
DSCAM	4mCPred	137	121	16
	4mCPred_SVM	137	122	15
	This study	137	126	11

More details of the prediction are presented in Table 4. Since the testing data in the case study only contains positive samples, there are only TP and FN counts in the results. For the dlk-1 case, 4mCPred has only one wrong prediction and the proposed method has made two false predictions out of 26 samples, while iDNA4mC and 4mCPred_SVM have 7 and 6 incorrect predictions respectively. For the DSCAM case, there are 137 4mC sites tested, and our proposed method has made 126 correct predictions (i.e., only 11 incorrect predictions). 4mCPred and 4mCPred_SVM have 16 and 15 false predictions, while iDNA4mC has made 67 false predictions. More detailed results can be found at the supplementary Additional file 3.

## Discussion

To improve the performance, we have focused on choosing more efficient features for 4mC site prediction, including extracting better sequence feature and feature selection before model learning. However, there are also some limitations in the study: first,the feature are mostly from the content of sequence, not the biological characters; second, the size of training data is limited.

In the future, we will continue to optimize our feature space with novel sequence features of important biological characteristics. Furthermore, we will expand the size of the benchmark datasets to enhance the model’s accuracy and generalization ability. Also, since the number of 4mC is much smaller than non-4mC sites in practical situations, the data imbalance will be considered in the next research. At last, we will apply our method to solve other sequence site prediction problems.

## Conclusions

The 4mC site prediction is a typical sequence site classification problem. The state-of-the-art research work have made some explorations, but their performance still needs improvement. For this purpose, we propose to construct a more effective feature space, integrating five types of sequence features, and suggest to use a novel learning algorithm with XGBoost based feature selection scheme. The results show that the feature selection improves the performance, and the prediction model outperforms the other three existing predictors in the independent tests and the cross-validations.

## Methods

Based on the benchmark datasets, this paper proposed a new sequence feature space and a machine learning algorithm with feature selection scheme. In the sequence encoding, five types of sequence features are integrated to form a 292-dimension feature space, representing both global and local sequence characteristics. Then a feature selection scheme is applied, by which feature importance score derived from the training process of the XGBoost machine is taken as the criterion of feature selection. Then an SVM prediction model is trained under the selected features and optimized by 10-fold cross-validation.

### Benchmark datasets

From the DNA 4mC database MethSMRT, Chen *et al* constructed the benchmark databases containing *Caenorhabditis elegans (C.elegans), Drosophila melanogaster (D.melanogaster), Arabidopsis thaliana (A.thaliana), Escherichia coli (E.coli), Geoalkalibacter subterraneus (G.subterraneus) and Geobacter pickeringii (G.pickeringii)* [23]. The benchmark datasets are obtained from Chen’s work. According to the reference, the 41-bit 4mC-centred DNA sequences were obtained from MethSMRT with a Modification QV threshold of 30. The CD-HIT software was used to remove the redundant positive samples. The same number of negative samples were selected randomly to construct a balanced dataset. The negative samples were also 41-bit cytosine-centered DNA sequences and were not detected by SMRT. To compare with the existing predictors, we use the same division of the datasets for independent tests. The summary of benchmark datasets is listed in Table 5.

**Table 5:** Summary of six benchmark datasets

Species	Positive Sample	Negative Sample	Total
*C.elegans*	1554	1554	3108
*D.melanogaster*	1769	1769	3538
*A.thaliana*	1978	1978	3956
*E.coli*	388	388	776
*G.subterraneus*	906	906	1812
*G.pickeringii*	569	569	1138

### Feature space construction

**Fig. 5:**
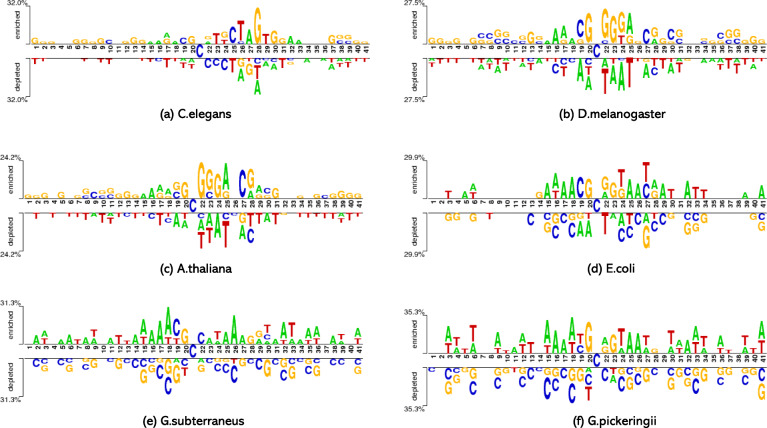
Sequence logos for DNA samples in the benchmark datasets

To visualize the difference between the positive and negative sequences, the sequence logos of all the six species are plotted using the web tool ‘two sample logos’ [39]. See Fig. 5. It is clear that the sequence characteristics are distinct among the six species; especially positions near the 4mC sites exhibit different patterns in positive and negative samples. In addition, the adjacent nucleotide and spectrum nucleotide across the entire sequence have specific patterns in different label groups. Thus an expanded feature space combining global and local patterns is good to construct accurate models for all the species. Among the existing methods, iDNA4mC only use nucleotide chemical property and frequency feature, which cannot extract the local adjacent nucleotide patterns; in 4mCPred and 4mCPred_SVM, the features mainly focus on the trinucleotide or dinucleotide nucleotide patterns, ignoring the spectrum nucleotide patterns. In this study, the feature space covers five types of features, one-hot 4-bit binary feature (OHB), sequential nucleotide frequency (SNF), k-nucleotide frequency (KNF), k-spectrum nucleotide pair frequency (KSNPF) and PseDNC. The OHB and SNF feature possess the information of the whole sequence and represent the global sequential properties, while KNF, KSNPF, and PseDNC features capture the local sequence patterns.

#### One-hot binary feature

The one-hot binary feature is the most widely used sequence representation feature. It converts each of the nucleotides in the DNA sequence into a 4-bit vector, which contains only one ‘1’. The length of the OHB feature is related to the number of nucleotide types and length of the sequence. Since the DNA sample sequence here is 41-bit and has four types of nucleotide, the one-hot binary feature is 164 bits. The encoding rules in this study are as follows: ‘A’-(1,0,0,0), ‘G’-(0,1,0,0), ‘T’-(0,0,1,0), ‘C’-(0,0,0,1). From the rule, it is obvious that the OHB feature is sparser than 2-bit or 3-bit binary features. But, the one-hot binary feature makes it more reasonable to calculate the importance sore for each dimension in feature space and to discover local motifs.

#### Sequential nucleotide frequency

The sequential nucleotide frequency, also known as nucleotide density, is the frequency that the corresponding nucleotide occurs before the current position. SNF is commonly used together with the binary encoding feature as a global density feature. For an n-bit long sequence, SNF calculates *n* values for each position in the sequence and produces an *n*-dimensional feature that starts with ‘1’. The SNF feature *d*_*i*_ is defined as:

1 $$ d_{i} =\frac{1}{\left | S_{i} \right |} \sum_{j=1}^{i}f(s_{j}), f(s_{j})=\left\{\begin{array}{ll} 1 & s_{j} = s_{i}\\ 0 & s_{j} \neq s_{i} \end{array}\right.  $$

where *S*_*i*_ denotes the length of sequence before the current position *i* and *s*_*i*_ is the nucleotide at position *i*. For example, a sequence like *‘AACGTACT’* can be converted into the SNF feature vector (1, 0.5, 0.33, 0.25, 0.2, 0.5, 0.28, 0.25).

#### k-nucleotide frequency

The k-nucleotide (k-mer) frequency is a classic concept in DNA sequence encoding. KNF feature is the frequency that adjacent k nucleotides occur in the whole sequence. The length of the KNF feature vector is 4^k^, determined by the parameter *k*. The calculation of KNF is as below:

2 $$ F\left(n_{1}n_{2}...n_{k}\right)=\frac{C\left(n_{1}n_{2}...n_{k}\right)}{S-k+1}  $$

where *n*_1_*n*_2_...*n*_*k*_ donates the adjacent k nucleotides and *n*_*i*_∈*(A, C, G, T)*. *F* and *C* is the feature value and total count of the adjacent nucleotides, while *S* is the length of sequence. When k = 1, the KNF is a vector like (*F*_*A*_, *F*_*C*_, *F*_*G*_, *F*_*T*_); when k =2, the KNF of a sequence is like (*F*_*AA*_, *F*_*AC*_, *F*_*AG*_, *F*_*AT*_, *F*_*CA*_, *F*_*CC*_, *F*_*CG*_, *F*_*CT*_, *F*_*GA*_, *F*_*GC*_,*F*_*GG*_, *F*_*GT*_, *F*_*TA*_, *F*_*TC*_, *F*_*TG*_, *F*_*TT*_) with a dimension of 4^2^ = 16.

#### k-spectrum nucleotide pair frequency

The KSNPF feature depicts the sequence context by calculating the frequency of k-spaced nucleotide pairs (e.g., AXXT is a two-spaced nucleotide pair, and CXXXG is a three-spaced nucleotide pair). Like the adjacent nucleotides pair above, the feature dimension of the KSNPF is 16 for each *k*. The calculation of this feature is as follows:

3 $$ F\left(n_{1}X...Xn_{2}\right)=\frac{C\left(n_{1}X...Xn_{2}\right)}{S-k-1}  $$

where *n*_1_*X*...*Xn*_2_ donates the k-spaced nucleotides pair and *n*_*i*_∈*(A, C, G, T)*.

#### PseDNC

As an essential sequence feature, PseDNC combines global and local structural properties and has been widely used in sequence site prediction problems [40]. For a DNA sequence, the PseDNC feature is a vector:

4 $$ F_{PseDNC} = \left [d_{1},d_{1},...d_{16}d_{16}...d_{16+\lambda} \right ]^{T}  $$

where,

5 $$ d_{k}=\left\{\begin{array}{ll} \frac{f_{k}}{\sum_{i=1}^{16}f_{i}+w\sum_{j=1}^{\lambda}\theta_{j}} & (1\leq k \leq 16)\\ \frac{w\theta_{\mu}-16}{\sum_{i=1}^{16}f_{i}+w\sum_{j=1}^{\lambda}\theta_{j}} & (16 < k \leq 16+\lambda) \end{array}\right.  $$

where *f*_*k*_ denotes the normalized frequency of two adjacent nucleotide pairs; *w* is the weight factor, and *θ* is the correlation factor of j-tier, representing the correlation of all j-tier from the sequence. The definition of *θ* is:

6 $$ \theta_{j}=\frac{1}{L-j-1}\sum_{i=1}^{L-j-1}\Theta_{i,i+j} (j=1,2,...,\lambda; \lambda <L)  $$

where *Θ* is the correlation function and given by:

7 $$ \Theta_{i,i+j} = \frac{1}{\mu }\sum_{u=1}^{\mu }\left [P_{u}(R_{i}R_{i+1}) - P_{u}(R_{j}R_{j+1}) \right ]^{2}  $$

where *μ* is the length of sequence; *P*_*u*_(*R*_*i*_*R*_*i*+1_) is the numerical value of the u-th DNA local property for the adjacent nucleotide pair *R*_*i*_*R*_*i*+1_ at position *i*. In this study, PseDNC feature is computed by a python package ‘repDNA’ [41] and the *λ* value is default to 3. The names of 38 DNA local properties utilized in the definitions here are detailed in the supplementary Table S1 of Additional File 1.

### Feature selection scheme

Feature selection can reduce the dimension of feature space and speed up the model training. A lot of feature selection strategies have been employed in machine learning [42]. In particular, a filter feature selection scheme has been used to improve the prediction performance. The filter feature selection scheme has two steps: first, F-score is calculated for each dimension in feature space according to the relevance between feature and label; second, a selection strategy called SFS is adopted to ascertain the feature subset. In this study, we proposed an embedded feature selection method also with two steps. However, we rank features with importance scores produced from the XGBoost training process [43] and select the top features with cross-validations.

In our method, XGBoost is the predefined classifier to analyze the feature importance. XGBoost has been proved to be an efficient tool in data science. In the training process, the XGBoost classifier calculates the feature importance score for each dimension based on the dimension location and the split efficiency in the boosting tree. In this study, XGBoost is implemented with a python package *`xgboost*^′^ of vision 0.90. The feature importance scores are obtained through the function *`get*_*score*^′^. According to the calculation method, the feature importance score has 5 types: *`weight*^′^, *`gain*^′^, *`cover*^′^, *`total*_*weight*^′^, *`total*_*gain*^′^ and here we use the default *`weight*^′^ importance score.

With the importance scores derived by the XGBoost classifier, feature dimensions are ranked from the highest to the lowest. Then the lower-ranked features are removed from the feature space one by one, and the feature subset performance is evaluated by 10-fold cross-validation with a support vector machine. The feature subset with the best performance is taken as the final feature space for 4mC prediction.

### Support vector machine

Support vector machine (SVM) is a popular machine learning classifier and has been proved to be more efficient than the other algorithms for DNA 4mC prediction in the state-of-the-art researches [25]. In this study, SVM is implemented by using the python package *`scikit*−*learn*(*vision*0.22)^′^ [44]. The kernel function of the SVM prediction model is set as a radial basis kernel function (RBF). The hyperparameter C and *γ* are optimized by a grid search with cross-validations and the search ranges are listed below:

8 $$ \left\{\begin{array}{ll} 2^{-5}\leq C \leq 2^{10} & step = 2\\ 2^{-15}\leq \gamma \leq 2^{2} & step = 2^{-1} \end{array}\right.  $$

With the output of the probability scores, the ROC curve can be plotted. The threshold of probability score is set as 0.5 to obtain the predicted label. Here, we compare SVM with other three traditional machine learning methods, such as Random Forest, Naive Bayes and Neural Network, and the results are reported in Table S3 of Additional File 1.

### Performance evaluation metrics

To compare with the existing predictors, the evaluation metrics in this study are consistent with the state-of-the-art methods, including Sensitivity(Sn), Specificity(Sp), Accuracy(ACC) and Matthews correlation coefficient(MCC). The definitions of these four metrics are as follows:

9 $$ \left\{\begin{array}{l} Sn = \frac{TP}{TP+FN} \times 100\%\\ \\ Sp = \frac{TN}{TN+FP} \times 100\\ \\ Acc = \frac{TP+TN}{TP+FN+TN+FP} \times 100\\ \\ Mcc = \frac{TP\times TN - FP\times FN}{\sqrt{(TP+FN)(TP+FP)(TN+FN)(TN+FP)}} \end{array}\right.  $$

Sn shows the model capability of identifying positive samples, while Sp tells the capacity of classifying negative samples; ACC is the prediction accuracy of all samples; MCC evaluates the overall performance of a predictor. In this study, the receiver operating characteristic(ROC) curve is also used to analyze model performance. The ROC curve is plotted in a coordinate graph where the x-axis is the false positive rate(1-Sp) and the y-axis is the true positive rate(Sn). The area under the curve(AUC) evaluates the classification performance, and larger AUC means better performance.

## Supplementary information

**Additional file 1** Supplementary tables. Table S1: The 38 physic-chemical property in PseDNC feature calculation of repDNA package. Table S2: The top 30 feature dimensions of feature importance score ranking in six species.

**Additional file 2** Feature importance scores. In Additional File 2, the importance scores for each feature dimension are reported. The feature scores are listed by the position number in all six species.s

**Additional file 3** Case study results. In Additional File 3, the 4mC-contained DNA sequence and the prediction results of four predictors are reported.

## Data Availability

The benchmark datasets analyzed during the current study are available in 4mCPred website (http://server.malab.cn/4mCPred/data.jsp). The data used in case study are collected from MethSMRT databse (http://sysbio.gzzoc.com/methsmrt/index.html). The three state-of-art predictors are available: iDNA4mC at (http://lin-group.cn/server/iDNA4mC.php), 4mCPred at (http://server.malab.cn/4mCPred/index.jsp) and 4mCPred_SVM at (http://server.malab.cn/4mcPred-SVM/). The source code and datasets in this study are freely available at https://github.com/Zhixun-Zhao/4mCPrediction.

## Abbreviations

4mC: DNA N4-methylcytosine(4mC)
OHB: one hot binary
SNF: sequential nucleotide frequency
KNF: k-nucleotide frequency
KSNPF: k-spectrum nucleotide pair frequency
ROC: the receiver operating characteristic curve
MCC: Matthews correlation coefficient
ACC: Accuracy
