# Supplementary material for: Accurate prediction of DNA N4-methylcytosine sites via boost-learning various types of sequence features
Source: BMC Genomics. 2020 Sep 11;21:627. doi: 10.1186/s12864-020-07033-8 (PMC7488740; doi:10.1186/s12864-020-07033-8)
Supplement: Supplementary file 1 — Additional file 1 Supplementary tables. Table S1: The 38 physic-chemical property in PseDNC feature calculation of repDNA package. Table S2: The top 30 feature dimensions of feature importance score ranking in six species. [file 12864_2020_7033_MOESM1_ESM.pdf]

Table S1. The 38 physicochemical property in PseDNC feature calculation of repDNA package.

Base stacking	Protein induced deformability	B-DNA twist
A-philicity	Propeller twist	Duplex stability: (freeenergy)
DNA denaturation	Bending stiffness	Protein DNA twist
Aida_BA_transition	Breslauer_dG	Breslauer_dH
Electron_interaction	Hartman_trans_free_energy	Helix-Coil_transition
Lisser_BZ_transition	Polar_interaction	SantaLucia_dG
SantaLucia_dS	Sarai_flexibility	Stability
Sugimoto_dG	Sugimoto_dH	Sugimoto_dS
Duplex tability (disruptenergy)	Stabilising energy of Z-DNA	Breslauer_dS
Ivanov_BA_transition	SantaLucia_dH	Stacking_energy
Watson-Crick_interaction	Dinucleotide GC Content	Twist
Tilt	Roll	Shift
Slide	Rise	

Table S2. The top 30 feature dimensions of feature importance score ranking in six species.

Rank	C.elegans	D.melanogaster	A.thaliana	E. coli	G.subterraneus	G.pickeringii
**1**	111	79	87	206	232	229
**2**	98	86	284	106	286	226
**3**	105	92	106	107	226	274
**4**	220	87	93	108	251	241
**5**	104	99	100	105	101	79
**6**	107	283	112	235	111	225

**7**	103	88	97	88	258	280
**8**	94	96	280	74	79	247
**9**	119	73	95	69	71	210
**10**	125	94	111	217	69	289
**11**	96	105	79	70	283	91
**12**	79	90	236	265	115	286
**13**	102	110	110	125	206	222
**14**	286	74	108	102	229	270
**15**	100	65	91	131	74	97
**16**	74	61	223	94	86	50
**17**	108	95	73	91	102	251
**18**	116	93	107	289	119	206
**19**	215	69	287	96	65	141
**20**	93	106	61	61	70	248
**21**	61	278	105	79	114	287
**22**	237	66	66	224	263	164
**23**	284	104	279	95	75	161
**24**	37	62	90	93	207	12
**25**	86	100	99	161	289	74
**26**	124	235	65	64	254	69
**27**	110	97	209	207	248	249
**28**	229	108	231	232	274	86
**29**	92	289	89	277	104	235
**30**	60	98	68	50	246	57

Table S3. Performance comparison of SVM and other machine learning methods

Machine learning method	species	Sn	Sp	ACC	MCC
Random Forest	C.elegans	0.731	0.731	0.731	0.462
	D.melanogaste	0.695	0.763	0.729	0.459
	A.thaliana	0.727	0.765	0.746	0.493
	E. coli	0.692	0.769	0.731	0.463
	G.subterraneus	0.767	0.717	0.742	0.484
	G.pickeringii	0.737	0.789	0.763	0.528
Naïve Bayes	C.elegans	0.692	0.75	0.721	0.443
	D.melanogaste	0.729	0.831	0.78	0.562
	A.thaliana	0.667	0.765	0.716	0.434
	E. coli	0.615	0.769	0.692	0.389
	G.subterraneus	0.717	0.7	0.708	0.417
	G.pickeringii	0.711	0.868	0.789	0.586
Neural Network	C.elegans	0.846	0.692	0.769	0.545
	D.melanogaste	0.763	0.805	0.784	0.568
	A.thaliana	0.788	0.811	0.799	0.598
	E. coli	0.808	0.808	0.808	0.615
	G.subterraneus	0.883	0.717	0.8	0.609
	G.pickeringii	0.868	0.842	0.855	0.711
SVM	C.elegans	0.942	0.789	0.865	0.74
	D.melanogaste	0.847	0.864	0.856	0.71
	A.thaliana	0.803	0.833	0.818	0.637
	E. coli	0.885	0.885	0.885	0.77
	G.subterraneus	0.917	0.817	0.867	0.74
	G.pickeringii	0.868	0.895	0.882	0.76
